# Supplementary material for: Generating correlated data for omics simulation
Source: PLoS Comput Biol. 2025 Sep 5;21(9):e1013392. doi: 10.1371/journal.pcbi.1013392 (PMC12422586; doi:10.1371/journal.pcbi.1013392)
Supplement: S1 Fig — (PDF) [file pcbi.1013392.s001.pdf]

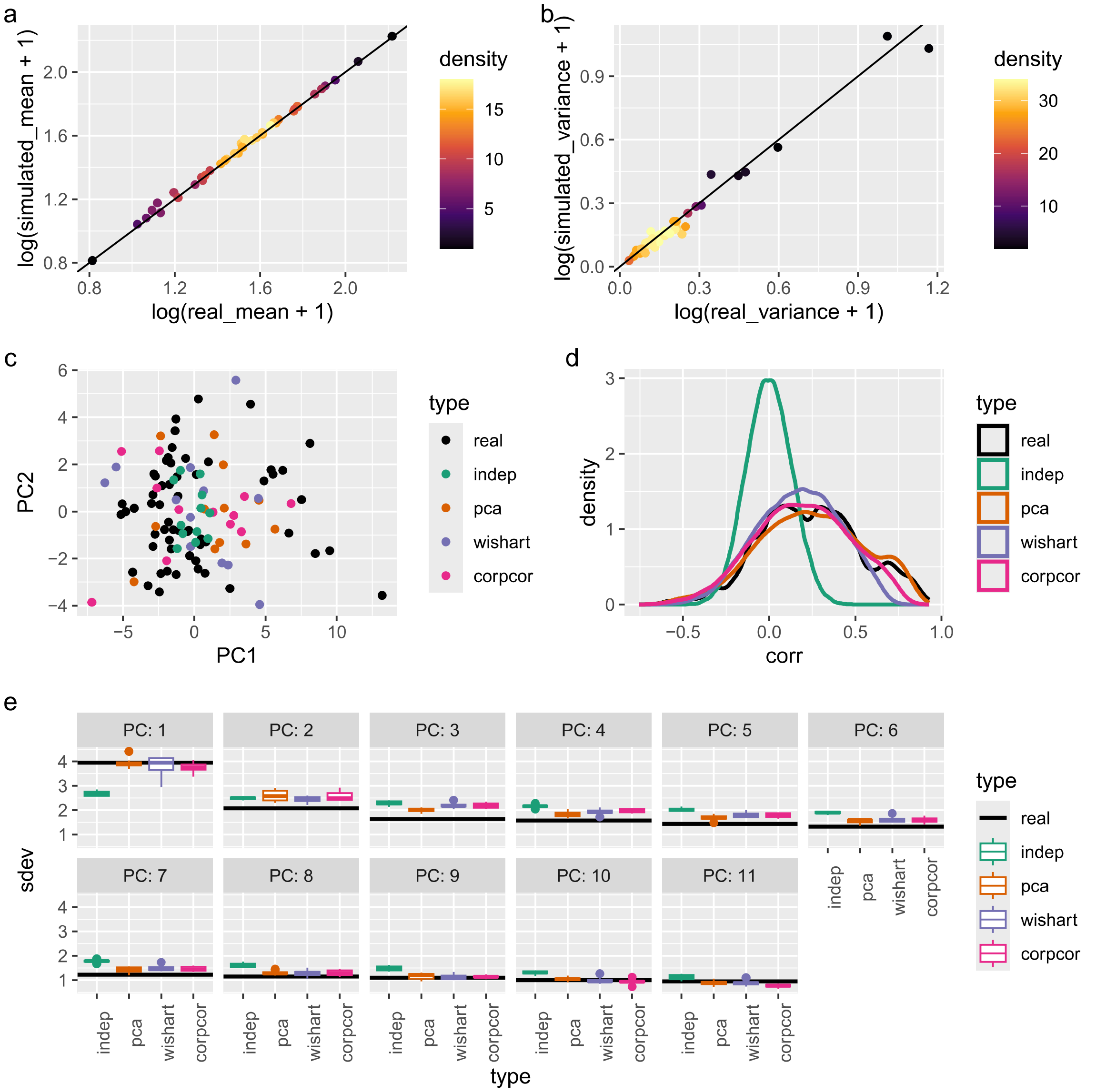

Figure 1: Comparison to plasma metabolomics data set, obtained from MetaboAnalyst (https://www.metaboanalyst.ca/MetaboAnalyst/upload/RocUploadView.xhtml). Methods were run using normal marginal distributions. Unlike other examples, this included more samples (59) than measured features (42 metabolites), demonstrating that these methods also work in that case. Data were log-scaled prior to simulation.
