# Supplementary material for: Generating correlated data for omics simulation
Source: PLoS Comput Biol. 2025 Sep 5;21(9):e1013392. doi: 10.1371/journal.pcbi.1013392 (PMC12422586; doi:10.1371/journal.pcbi.1013392)
Supplement: S2 Fig — (PDF) [file pcbi.1013392.s002.pdf]

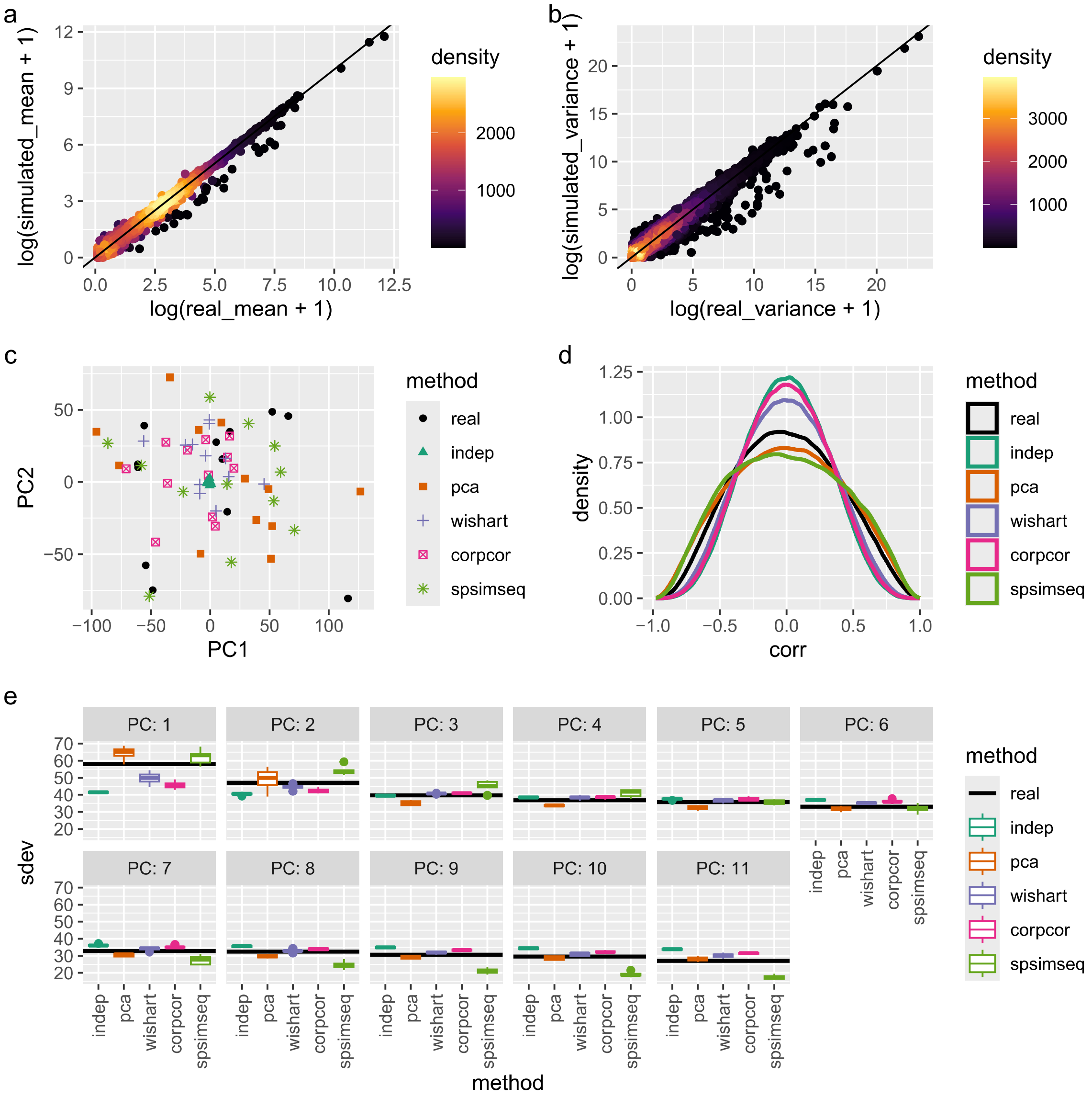

Figure 2: Comparison to real data run on a fly whole body data set from GSE81142. (a-b) Comparison of gene (a) mean expression and (b) variance, log-scaled in real and PCA simulated data. The line of equality is marked in black. Points are colored according to the density of points in their region. (c) Quantile-quantile plot comparing correlation values of gene pairs from real data and simulated data (both with and without dependence). Genes with at least 30 reads were used. Values on the diagonal line indicate a match between the simulated and real data sets. (d) Projections onto the top two principal components of the real data set for both real and simulated data. All 8 simulations (96 samples for each simulation) shown. (e) Principal component analysis was performed on all data sets and the variance captured by the top components is shown. Unlike (d), these components were fit from each data set considered separately instead of reusing the weights from the real data.
