# Supplementary material for: Generating correlated data for omics simulation
Source: PLoS Comput Biol. 2025 Sep 5;21(9):e1013392. doi: 10.1371/journal.pcbi.1013392 (PMC12422586; doi:10.1371/journal.pcbi.1013392)
Supplement: S1 Text — (PDF) [file pcbi.1013392.s004.pdf]

Generating correlated data for omics simulation

Supplemental Methods

Running SPsimSeq

The R package SPsimSeq (Assefa, Vandesompele, and Thas 2020) provides a dedicated RNA-seq and single-cell RNA-seq simulator using a Gaussian copula approach to simulate gene dependence. We ran SPsimSeq using the following options:

```
    n.sim = 1,
    s.data = input,
    n.genes = input |> nrow(),
    batch.config = 1,
    group.config = 1,
    pDE = 0,
    tot.samples = 100,
    model.zero.prob = FALSE,
    genewiseCor = TRUE,
    log.CPM.transform = TRUE,
    result.format = "list",
    return.details = TRUE,
    verbose = TRUE,
```

This produces bulk RNA-seq data with no batching or group effects or differentially expressed (DE) genes. Raw count values provided as the input matrix. For dataset GSE151565, only the ZT0 timepoints were used. This is the same set of samples that were used in our simulation to determine the correlation structure (copula).

SPsimSeq was not run in the two example applications, DESeq2 and CYCLOPS, due to substantial differences in how it generates DE across samples compared to our method. DE in SPsimSeq is done by mimicking a random set of detected DE genes in the reference data set. SPsimSeq does not report either the group mean values or the log fold changes used for these genes, so we are not able to modify our own simulation to have the same set of DE genes at the same effect sizes.

Running vine copula

We used the rvinecopulib package to fit and generate random data from a vine copula distribution. This package is a R wrapper of the high-performance vinecopulib C++ package. This package expects data scaled to the range of [0, 1]. Therefore, we chose not to use the real RNA-seq count data and instead generated correlated random number as input which follow a multivariate normal distribution with covariance matrix constructed with a randomly generated singular vector matrix and singular values 1, 2, …, 100. These values were then scaled

to the interval [0,1] by the `pnorm` function. A vine copula was fit using just the "guassian" family. While rvinecopula supports multi-threaded operation, we ran using a single thread as with the other methods.

Running mvrnorm

To assess a simple multivariate normal distribution simulation option, we ran the `mvrnorm` function from the MASS R package. We set means to be the mean expression of each gene and used the sample covariance matrix as the `Sigma` parameter.

Running mvnfast

The `mvnfast` R package provides a faster implementation of the multivariate normal distribution random vector generator function, called `rmvn`. As for `mvrnorm`, we set means to be the mean expression of each gene and used the sample covariance matrix as the `Sigma` parameter. One core was used for a fair comparison to the other methods, though `mvnfast` does support multithreaded execution. Note that `mvnfast` has an option for using a provided pre-computed Cholesky decompositoin of the covariance matrix instead of producing it itself. Since Cholesky decomposition is the primary time-sink, using this option could likely considerably speed up the execution time if such a decomposition is known. We did not use this option since the comparison is for a generic covariance matrix, where the Cholesky decomposition is not known a priori. This contrasts to our implementation which avoids Cholesky decomposition by using only covariance matrices of a specific form.

Assefa, Alemu Takele, Jo Vandesompele, and Olivier Thas. 2020. "SPsimSeq: Semi-Parametric Simulation of Bulk and Single-Cell RNA-sequencing Data." *Bioinformatics* 36 (10): 3276–78.
