# Supplementary material for: Generating correlated data for omics simulation
Source: PLoS Comput Biol. 2025 Sep 5;21(9):e1013392. doi: 10.1371/journal.pcbi.1013392 (PMC12422586; doi:10.1371/journal.pcbi.1013392)
Supplement: Attachment — Submitted filename: 20250305.pdf [file pcbi.1013392.s005.pdf]

Summary

"Generating Correlated Data for Omics Simulation" offers a pedagogical introduction Gaussian copulas, written to encourage their use in benchmarking. Three methods from the diagonal-plus-low-rank covariance estimation literature are discussed and have been included in an accompanying R package. After reviewing methods, the manuscript describes how to evaluate the quality of a fitted simulator w.r.t. a reference dataset. Two illustrative case studies are given. The first demonstrates elevated FDR of the popular DESeq2 method when features are dependent. The second shows that a method for estimating circadian time improves in the presence of dependence.

Evaluation

The importance of gene-gene dependence is often overlooked in omics simulations, and this manuscript provides a readable discussion of the topic. The methods that it explains are technically sound and the case studies are authentic. My concerns are related to accessibility for the intended audience, the level of precision in the case studies, and the extent to which some claims in the abstract/introduction are justified.

Major

1. One of the selling points of the manuscript is that it provides a practical introduction to copulas, a topic that is usually only covered in graduate-level multivariate statistics courses. This could be extremely valuable, considering that many researchers are self-trained in statistics (and yet expected to critique others' simulations/write them for their own analysis). Perhaps I am misunderstanding the intended audience, but I am concerned that some readers will get lost in the methodological explanations. For example, will readers understand the phrase "marginal distribution" or the notation e_i for i^{th} standard basis vector without any definitions? I understand that a good paper should challenge readers to learn more, but I nonetheless encourage the authors to read through carefully and ask whether any language could be adjusted to avoid jargon and broaden reach.

2. There are many important details missing from the case studies. Most importantly:

   - What preprocessing was applied? In omics data, it is common to apply filtering or variable transformations, but I couldn't find information about this. Related: I couldn't find the data dimensionality and only realized the data were centered/scaled when $e_i^\top \hat{\Sigma} e_i \approx 1$ was mentioned in the Spiked Wishart section.
   - It wasn't clear what types of marginal models were being applied (Normal, Negative Binomial?) or how they were being estimated (maximum likelihood?). Why were they chosen for the particular case studies, among all the possible options?

3. This is problematic because the abstract and introduction highlight how copulas can be used with "discrete (binary ordered categorical, Poisson, negative binomial), continuous (Normal) or with an empirical distribution," but from the case studies, I left with the impression that they might have all been normal marginals fitted to $\log(1 + x)$-transformed data. If a wider range of marginals are applied, then this should be clearly described to back up the claims in the abstract/introduction. Otherwise, I think the points about flexibility need to be toned down, so readers don't get disappointed later.

4. It's unclear whether the "Comparison with real data" section is enough to warrant application in the following case studies. In practice, shouldn't users still check the quality of the simulated data in each application? How can you be sure that the case studies use simulated data that actually match the reference data they are fitted from? Are those data similar enough to the data from the earlier section?

Minor

1. For the DESeq2 case study, it seems worthwhile to add references to some literature discussing miscalibration among common differential analysis methods. The first that comes to mind is [2, 1], though I know there are others. These references might be especially useful because I suspect the first reaction of many readers may be, "Does this mean I can't use DESeq2?"

2. In the CYCLOPS case study, is some dependence being induced between samples through the marginal models? To interpret temporal trends between samples, don't the simulated data need to be temporally dependent? This seems to contradict the "features measured in each of n independent samples" setup at the start of the Results. My understanding is this requirement of independence across samples is not necessary if the marginal models can make use of covariate information.

3. In the "Comparisons with real data," there is some discussion about how the density of pairwise correlations (Fig 1d) either underestimated or "overshot" the observed density. There are correlation estimators with more precisely tunable regularization parameters, which could be used to exactly match the densities. C.f., the CovTools package [3] and discussion in this tutorial [4]. I don't think it's necessary to modify the experiment, since the focus is on the three implemented covariance estimators, but I wanted to make sure the authors were aware that the density-matching issue is solvable.

4. I was able to install the package and run the example on the GitHub page very easily. The README is well-written. Perhaps the authors could consider submitting to CRAN or Bioconductor.

5. There are still a few (not many) typos remaining, e.g., signular and sepctral.

References

[1] Stijn Hawinkel, Federico Mattiello, Luc Bijnens, and Olivier Thas. A broken promise: microbiome differential abundance methods do not control the false discovery rate. *Briefings in bioinformatics*, 20(1):210–221, 2019.

[2] Keegan Korthauer, Patrick K Kimes, Claire Duvallet, Alejandro Reyes, Ayshwarya Subramanian, Mingxiang Teng, Chinmay Shukla, Eric J Alm, and Stephanie C Hicks. A practical guide to methods controlling false discoveries in computational biology. *Genome biology*, 20:1–21, 2019.

[3] Kyoungjae Lee and Kisung You. Covtools: Statistical tools for covariance analysis. `http://dx.doi.org/10.32614/CRAN.package.CovTools`.

[4] Kris Sankaran, Saritha Kodikara, Jingyi Jessica Li, and Kim-Anh Lê Cao. Chapter 3 Multivariate Power Analysis — Simulation for Microbiome Analysis. `https://krisrs1128.github.io/microbiome-simulation/multivariate-power-analysis.html`.
