# Supplementary material for: Generating correlated data for omics simulation
Source: PLoS Comput Biol. 2025 Sep 5;21(9):e1013392. doi: 10.1371/journal.pcbi.1013392 (PMC12422586; doi:10.1371/journal.pcbi.1013392)
Supplement: Attachment — Submitted filename: Review Comments.pdf [file pcbi.1013392.s006.pdf]

In this article, the authors have described three approaches for “quickly” generating omics-scale data with correlated measures which mimic three datasets. The authors made an attempt to describe the three methods and they illustrated the performances of the three methods on (1) a mouse cortex RNA-seq dataset, (2) a DESeq2 application, and (3) a CYCLOPS application.

Overall, the structure of the article is clear. However, my overall evaluation of this article is that it has not met the standard for academic publication. There is obviously room for improvement and I encourage the authors to try making a major revision on their own first before resubmitting it.

For my review, I was hesitant between rejection and major revision, but chose rejection in the end. The following are some major problems I have found.

1. The authors’ description of their method has lots of problems. It has confusing descriptions and notations and is difficult to understand. For example:
    (1) The authors use the terms “normalizing” and “normalization” to mean applying the inverse standard Gaussian CDF to uniform random variables to convert into normal random variables. This is confusing since the commonly used meaning of “normalizing” and “normalization” is to convert some value to another scale, but not necessarily a normal random variable, e.g., total library size normalization or CPM normalization.
    (2) The authors did not distinguish between the parameters of the statistical models and the parameter estimates from the data.
    (3) The authors seem to use the notation Z to represent different objects. Sometimes it is a p-dimensional random vector. Sometimes it is a p by n matrix.
    (4) In the Methods Section, the authors did not give enough context before describing the methods. I suggest they move this part to the front of the article or include more context.
    (5) When describing the PCA method, the authors state V = Z^T Z / (n-1) is the covariance matrix of Z. However, this is incorrect if Z is p by n.
    (6) It is unclear what is the meaning of “reference dataset”.
2. The authors described three methods and included all of them in their R package. However, the last method from another R package proposed by other people. It doesn’t seem appropriate to directly use the same method and repackage it to be one’s own.
3. For DESeq2 application, Figure 2 shows the result. However, the performances across independence, PCA, Wishart, and corpcor seem to be the same, with the authors’ proposed methods have more variance. Therefore, it is unclear why the authors’ methods are better.
4. For CYCLOPS application, the authors did not give enough explanations on why corpcor is worse than PCA and Wishart.
5. In general, the authors did not give guidance on how to choose among their three described methods.
6. There are many types of omics datasets. Do their methods apply to all of them?

7. The authors have only compared their methods to independence. It is not surprising the methods that consider correlations have better performance. The authors should also compare their method to not other methods that consider correlations.
8. The authors state that their methods can compute the correlations quickly. They should describe this advantage in more details and include some examples to illustrate.

Overall, there are lots of room to improve and I hope the authors can make some big improvements of this article based on the current version.
