# Supplementary material for: Generating correlated data for omics simulation
Source: PLoS Comput Biol. 2025 Sep 5;21(9):e1013392. doi: 10.1371/journal.pcbi.1013392 (PMC12422586; doi:10.1371/journal.pcbi.1013392)
Supplement: Attachment — Submitted filename: plos_comp_bio_reviewer_response.docx [file pcbi.1013392.s007.docx]

Dear reviewers and editors,

Thank you for considering our manuscript and for the extensive feedback. We have made significant revisions to address concerns. Major additions include: performing comparison to the real reference data sets for all real, reference data used in the example applications; benchmarking to support our performance claims; comparisons of our method to another simulator (SPsimSeq) that includes dependency; and an improved focus of the manuscript on the specific challenge that our methods address (the choice of covariance matrix and efficiency). In addition, many smaller changes have been made. We believe that these changes have substantially improved the paper, and we thank you for the opportunity to revise.

Below, please find our responses (in black) to reviewer points (in red).

Reviewer 1:

**Major:**

1. **One of the selling points of the manuscript is that it provides a practical introduction to copulas, a topic that is usually only covered in graduate-level multivariate statistics courses. This could be extremely valuable, considering that many researchers are self-trained in statistics (and yet expected to critique others’ simulations/write them for their own analysis). Perhaps I am misunderstanding the intended audience, but I am concerned that some readers will get lost in the methodological explanations. For example, will readers understand the phrase “marginal distribution” or the notation ei for ith standard basis vector without any definitions? I understand that a good paper should challenge readers to learn more, but I nonetheless encourage the authors to read through carefully and ask whether any language could be adjusted to avoid jargon and broaden reach.**

**We agree with this concern and have significantly revised the structure of our manuscript. We now devote our main text to describe the primary problem that our methods address: the selection of a covariance matrix. Previously, this difficulty was not emphasized despite it being what separates our methods from other copula-based approaches. General descriptions of the copula approach have been moved to Methods and are now intended to act more as a reference material than as a tutorial.**

**We also introduce a new figure (Fig 1a) that provides a diagram overview of the method, and demonstrates the difficulty of selecting covariance matrices, showing how the most obvious choice of covariance matrix (the sample covariance matrix) is inadequate.**

2. **There are many important details missing from the case studies. Most importantly:**
   a. **What preprocessing was applied? In omics data, it is common to apply filtering or variable transformations, but I couldn’t find information about this. Related: I couldn’t find the data dimensionality and only realized the data were centered/scaled when** $\boldsymbol{e}_{\boldsymbol{i}}^{\boldsymbol{T}} \boldsymbol{\Sigma}\mathbf{e}_{\mathbf{i}}$ **was mentioned in the Spiked Wishart section.**
   b. **It wasn’t clear what types of marginal models were being applied (Normal, Negative Binomial?) or how they were being estimated (maximum likelihood?). Why were they chosen for the particular case studies, among all the possible options?**

**We apologize for these oversights. The RNA-Seq examples are all using a negative binomial marginal, fit using DESeq2. Since DESeq2 expects unnormalized, raw count data, we did no preprocessing of read counts. A normalization step (which we now refer to as ‘normalizing transformation’) later happens in the Gaussian copula step, which we describe now in the Methods section. It is this transformation that causes the equation** $\boldsymbol{e}_{\boldsymbol{i}}^{\boldsymbol{T}} \boldsymbol{\Sigma}\mathbf{e}_{\mathbf{i}}\boldsymbol{=1}$ **to hold. We have added more details to the manuscript for each of the example applications, for example:**

**All data were simulated with negative binomial distributions fit by DESeq2 on unnormalized read counts.**

**Lastly, our newly added metabolomics data set was log-scaled prior to use as a reference data set.**

3. **This is problematic because the abstract and introduction highlight how copulas can be used with “discrete (binary ordered categorical, Poisson, negative binomial), continuous (Normal) or with an empirical distribution,” but from the case studies, I left with the impression that they might have all been normal marginals fitted to log (1 + x)-transformed data. If a wider range of marginals are applied, then this should be clearly described to back up the claims in the abstract/introduction. Otherwise, I think the points about flexibility need to be toned down, so readers don’t get disappointed later.**

**All of the listed marginal families have been implemented in our software package and can be specified by the user. To better demonstrate this flexibility, we have added a new example using normal marginals on a log-transformed metabolomics data set (see Figure S1). This is in addition to the negative binomial marginal examples (which we refer to as “DESeq2” marginals since they also additionally contain a library size term in the model, meaning that individual samples have negative binomial marginals but that across samples the distributions may not be negative binomial if library size varies). Together, these examples demonstrate that our methods do not depend upon the marginal distribution.**

4. **It’s unclear whether the “Comparison with real data” section is enough to warrant application in the following case studies. In practice, shouldn’t users still check the quality of the simulated data in each application? How can you be sure that the case studies use simulated data that actually match the reference data they are fitted from? Are those data similar enough to the data from the earlier section?**

**We agree that this was inadequate. We have updated the manuscript to contain in Supplemental additional comparisons to real data sets for all reference data sets used in the examples. See Figure S2, S3. In addition to the one comparison in the main manuscript, this means that all reference data sets used in case studies have been checked for quality, all with similar results.**

**Minor**

1. **For the DESeq2 case study, it seems worthwhile to add references to some literature discussing miscalibration among common differential analysis methods. The first that comes to mind is [2, 1], though I know there are others. These references might be especially useful because I suspect the first reaction of many readers may be, “Does this mean I can’t use DESeq2?”**

**We agree that miscalibration is not a novel finding and is not unique to DESeq2. Moreover, our primary interest is in the change in variance across dependence simulations, while miscalibration occurs even in simulations without dependence.**

**We have updated the manuscript to add the following note to the reader on this in Discussion:**

**We note that DESeq2’s observed anti-conservative nature is most present at very low FDR thresholds and was typically associated with only a single digit number of false positive genes. Moreover, more modern methods to control FDR are compatible with DESeq2 and have been shown to be appropriately conservative (Korthauer et al. 2019).**

2. **In the CYCLOPS case study, is some dependence being induced between samples through the marginal models? To interpret temporal trends between samples, don’t the simulated data need to be temporally dependent? This seems to contradict the “features measured in each of n independent samples” setup at the start of the Results. My understanding is this requirement of independence across samples is not necessary if the marginal models can make use of covariate information.**

**This is an important point, so we apologize for the confusion. No, in all of our simulations, samples are independent of each other, conditional on group or time. That is, the mean values of the marginal distributions are functions of time (or group, in the DESeq2 application), but each sample is a random draw taken independently of the others from this distribution. To an observer who knew the ground truth mean and variance parameters, the read counts of one sample would tell them nothing about read counts of another sample.**

**For the CYCLOPS application, this reflects the typical assumptions of the data sets to which it is applied. For example, this method has been used on RNA-seq samples from GTEx, where each sample is a postmortem dissection of a tissue from separate individuals. In this case, we assume that each individual is independent of the others but that each sample might have some dependence upon time of day. We added the following clarification to the CYCLOPS application section:**

**We note that, unlike many timeseries methods that consider correlation across time points, the dependence here is between genes within the same sample and no dependence between samples or timepoints is generated. This reflects scenarios where CYCLOPS is used, for example, post-mortem biopsy samples of unrelated individuals collected at different times (Li et al. 2024).**

3. **In the “Comparisons with real data,” there is some discussion about how the density of pairwise correlations (Fig 1d) either underestimated or “overshot” the observed density. There are correlation estimators with more precisely tunable regularization parameters, which could be used to exactly match the densities. C.f., the CovTools package [3] and discussion in this tutorial [4]. I don’t think it’s necessary to modify the experiment, since the focus is on the three implemented covariance estimators, but I wanted to make sure the authors were aware that the density-matching issue is solvable.**

**Thank you for this interesting point. We have added discussion to our introduction about a greater variety of covariance matrix estimation methods, including the one referenced by CovTools for their adaptive method. Since the tutorial linked seems to rely on source code that is not currently publicly available, it is a little more challenging to comment on their methods. In particular, it looks like that although they use the CovEst.adaptive method for simulation, when we run it on other data sets, we generally get a matrix that is not positive semi-definite. This makes it challenging to use for simulation (which to my knowledge requires a positive semi-definite covariance matrix when generating multivariate normal values) and we do not know what solution to this the code of the provided tutorial uses. We therefore were unable to provide a more direct comparison with this method and our own, though this would certainly be of interest.**

4. **I was able to install the package and run the example on the GitHub page very easily. The README is well-written. Perhaps the authors could consider submitting to CRAN or Bioconductor.**

**Thank you for the kind words and for checking that our package works. We are currently working on getting it submitted to CRAN based off your suggestion.**

5. **There are still a few (not many) typos remaining, e.g., signular and sepctral.**

**These have been corrected and our manuscript proofread carefully.**

**Reviewer 2:**

1. **The manuscript describes a data simulation approach. Could you provide a clear rationale for this specific goal? Also, the authors demonstrate the covariance matrix decomposition into diagonal and low-rank components. Can you show what the hypothesis behind it. More specifically, how does this decomposition compare to other methods of modeling correlation structures in RNA-seq data?**

**We agree that this motivation was lacking. We have revised the manuscript substantially to better address this and to highlight that our methods are innovating about the choice covariance matrix. We have added a new figure (Fig 1 b) demonstrating why the sample covariance matrix is an inadequate choice, which motivates why we need an alternative like ours. We attempt to provide better intuition for the meaning of the diagonal and low-rank parts (specifically that the diagonal component captures an “injection” of extra independence above what is measured by the low-rank covariance matrix).**

**The other motivation for our proposed methods is that this specific form admits highly efficient generation of simulated data. We added the following text to Methods to explain this simulation scheme:**

**Generating data for** $\boldsymbol{\Sigma}_{\boldsymbol{sim}}$ **of this form is highly efficient. We use two basic facts about the multivariate distribution. First, if A is a matrix and** $\boldsymbol{u \sim N(0,}\boldsymbol{\Sigma}\boldsymbol{)}$ **then** $\boldsymbol{Au \sim N(0, A}\boldsymbol{\Sigma}\boldsymbol{A}^{\boldsymbol{T}}\boldsymbol{)}$**. Second, if** $\boldsymbol{u \sim N(0,}\boldsymbol{\Sigma}_{\boldsymbol{1}}\boldsymbol{)}$ **and** $\boldsymbol{v \sim N(0,}\boldsymbol{\Sigma}_{\boldsymbol{2}}\boldsymbol{)}$**, then** $\boldsymbol{u+v \sim N(0,}\boldsymbol{\Sigma}_{\boldsymbol{1}}\boldsymbol{+}\boldsymbol{\Sigma}_{\boldsymbol{2}}\boldsymbol{)}$**. Therefore, given matrices D, U, W and** $\boldsymbol{u \sim N(0,}\boldsymbol{I}_{\boldsymbol{k}}\boldsymbol{)}$ **and** $\boldsymbol{v \sim N(0,}\boldsymbol{I}_{\boldsymbol{p}}\boldsymbol{)}$ **(where** $\boldsymbol{I}_{\boldsymbol{k}}\boldsymbol{,}\boldsymbol{I}_{\boldsymbol{p}}$ **are the** $\boldsymbol{k\times k, p\times p}$ **identity matrices), then** $\boldsymbol{Dv+UW u \sim N}\left( \boldsymbol{0,}\boldsymbol{D}^{\boldsymbol{2}}\boldsymbol{+U}\boldsymbol{W}^{\boldsymbol{2}}\boldsymbol{D}^{\boldsymbol{T}} \right)\boldsymbol{=N(0,}\boldsymbol{\Sigma}\boldsymbol{)}$ **as desired.**

**In contrast, when given an arbitrary** $\boldsymbol{\Sigma}$ **to generate a random value in** $\boldsymbol{N}\left( \boldsymbol{0,}\boldsymbol{\Sigma} \right)$ **one must compute a matrix V such that** $\boldsymbol{V}\boldsymbol{V}^{\boldsymbol{T}}\boldsymbol{=}\boldsymbol{\Sigma}$**. Then, using** $\boldsymbol{v \sim N}\left( \boldsymbol{0,I} \right)$**, we have** $\boldsymbol{Vv \sim N}\left( \boldsymbol{0,}\boldsymbol{\Sigma} \right)$ **as desired. However, computing V is done using a Cholesky decomposition or eigenvector decomposition, both of which are computationally expensive when** $\boldsymbol{\Sigma}$ **is large.**

**We also emphasize this advantage further in Results and have added a benchmarking of performance of methods to demonstrate this concretely. These benchmarks (Figure 1 c-d) shows over 100x run time and 20x memory use compared to our methods when simulating 32,000 genes (for SPsimSeq or mvrnorm) or just 2,000 genes (for a vine copula method). Memory requirements, in particular, are an obstacle to alternative approaches on typical machines. This emphasizes why our choices are practical and convenient.**

**We have also added the SPsimSeq method to each of our RNA-seq examples to compare the fidelity of our simulations with this method. See Fig 2, S2 and S3. This shows our PCA method is most similar to SPsimSeq and that all methods are improvements over independent data. We also correct and expand our description of SPsimSeq in Discussion, where we previously incorrectly said it used a network based approach to covariance.**

2. **For the introduction section, could you include more related works on probabilistic models and generative models that are relevant to data simulation and benchmarking? Providing a broader context on these approaches would strengthen the foundation of the study.**

**We apologize for this omission and have added to the introduction discussion of copula methods in omics and omics simulation and also of covariance matrix estimation methods. We believe that this provides better context for the reader and highlights that there are many alternative approaches that could be taken. We added the following text to Introduction:**

**There is a large body of literature on determining covariance matrices for various purposes. Shrinkage methods (Schäfer and Strimmer 2005; Opgen-Rhein and Strimmer 2007) improve estimates and guarantee that the estimated matrix is well-conditioned (and hence invertible). Sparse matrix methods Cai and and (2011) produce a matrices where many of the pairwise correlations are zero. Similarly, other methods produce a sparse estimate of the inverse of the covariance matrix (Meinshausen and BÃ¼hlmann 2006), which imparts pairwise independence of some variables conditional on all the others.**

**Copula approaches are flexible and have a long history (Sklar 1973) of use in fields such as economics (MacKenzie and Spears 2014) More recently, copula approaches have been used for modelling or classification in multi-omics fields (He et al. 2020; Ma, Davis, and Ho 2023) and metagenomics (Deek and Li 2023). Projects simulating omics with copulas include SPsimSeq (Assefa, Vandesompele, and Thas 2020) and scDesign2 (Sun et al. 2021). Some methods have employed the “vine copula” approach in the context of single-cell or spatial omics simulation (Fuetterer, Schollmeyer, and Augustin 2019; Song et al. 2024). This provides an alternative approach for choosing a copula which decomposes it into a set of 2-dimensional dependencies, however it comes at significant computational cost when done at the omics-scale. SeqNet uses instead a network approach for gene dependence (Grimes and Datta 2021).**

**We also added some related comments in Discussion:**

**While we describe one special form of**  $\hat{\boldsymbol{\Sigma}}$ **where generation is highly efficient, there exist many other possible covariance matrix forms. Block diagonal covariance matrices are one such example which can efficiently represent even high-dimensional data (Devijver and Gallopin 2018) and lend themselves to efficient simulation. Sparse matrix methods (Bien and Tibshirani 2011; Bickel and Levina 2008; Cai and Weidong 2011) yield exactly zero correlation between some variables, but it is less clear how to simulate data from these efficiently. Iterative sampling methods (Chow and Saad 2014) are one approach that could exploit the sparse nature and may even extend to sparse inverse covariance methods (Meinshausen and Bahlmann 2006).**

**While the study compares simulated data to real datasets, the validation metrics primarily focus on variance and correlation structure. Could you provide additional validation using biological benchmarks (e.g., differential analysis, functional enrichment) could further support the biological relevance of the simulated datasets.**

**Thank you for raising this point. Unfortunately, we are not able to compare differential behavior with the real data sets. This is because our differential expression is entirely parametrically added in to the simulation and is not based off the original data sets. We selected data sets that provided a relatively large number of replicates (12) without strong differential expression between any groups, which limits significantly the choice of possible experiments, and we struggled to find such a dataset that had a ‘typical’ amount of differential expression (i.e., neither no genes differential nor having nearly every gene differential). We note that parametrically creating differential expression is also done by alternatives, such as SPsimSeq, which randomly chooses a subset of genes to make DE and randomly assigns log fold changes for those genes. This has the benefit of allowing the amount of DE to be easily modified.**

3. **The results indicate increased variance in DESeq2 outcomes when using correlated data, but the practical implications for differential expression analysis are not fully explored. What does this increased variance represent?**

**We interpret this as saying that, in more realistic data sets, DESeq2 is more likely to produce multiple false positives simultaneously, despite having the same average number of false positives. However, we emphasize that this was occurring primarily at quite low false discover rate cutoffs and DESeq2 was close to well-calibrated at more typical cutoffs like 0.1. Our primary interest in this is not the direct impact on differential expression analysis but rather on the benchmarking of this. We believe that this increased variance is sufficiently important that it motivates the need for benchmarking to include simulated dependence of genes. We added the following to Discussion:**

**We note that DESeq2’s observed anti-conservative nature is most present at very low FDR thresholds and was typically associated with only a single digit number of false positive genes. Moreover, more modern methods to control FDR are compatible for DESeq2 and have been shown to be appropriately conservative (Korthauer et al. 2019).**

4. **By comparing with the real datasets, the authors compared the independent genes with the dependent genes with various methods. But none of the existing simulation approach has been compared. Can you benchmark with existing methods and show your simulation performances, e.g scDesign, ZINB-WAVE, etc.?**

**Thank you for raising this important point. We now include comparisons to SPsimSeq in the figures where we compare fidelity (Fig 2, S1, S2) to the reference real data sets. This shows that SPsimSeq performs comparably to our own, particularly similarly to our PCA method. We also added benchmarking showing run-time performance and memory usage of our methods, SPsimSeq, multivariate normal random values using the mvrnorm function, and a vine copula method which has been used in two relatively recent simulators (Fig 1b). This benchmarking demonstrates a large advantage in run time and memory usage for our methods. The vine copula approach does not scale at all to the gene counts we use in these benchmarks, so we do not run it on the example applications. The mvrnorm data is insufficiently realistic for actual application due to assuming a normal distribution and so was not used in example applications.**

5. **What is your conclusion for this three different methods? It seems like there is no consistent best methods across different datasets.**

**Thank you for the suggestion. We have added some better guidance for this to Discussion, though the choice is ultimately application-specific:**

**We observe that our PCA method generally overshoots the level of correlation in the real data set. The corpcor method typically undershoots. The spiked Wishart method also undershoots but less so than the corpcor method and therefore we believe represents the best starting point for users. However, we emphasize that in any application, the users should compare their simulated data to the reference data set and this can be used to determine the best method for the specific application at hand, which could vary depending on data set.**

**Reviewer 3:**

1. **The authors’ description of their method has lots of problems. It has confusing descriptions and notations and is difficult to understand. For example:**
1. **The authors use the terms “normalizing” and “normalization” to mean applying the inverse standard Gaussian CDF to uniform random variables to convert into normal random variables. This is confusing since the commonly used meaning of “normalizing” and “normalization” is to convert some value to another scale, but not necessarily a normal random variable, e.g., total library size normalization or CPM normalization.**

**We have revised this to now use the terminology “normalizing transform”. We were unable to find an exact match of existing terminology to our use case, since “normalizing transform” most frequently refers to a quantile-based approach instead of the distribution fitting approach that we employ, but this seems to be the most similar terminology available. Normalization is now used just to refer to CPM normalization or similar.**

2. **The authors did not distinguish between the parameters of the statistical models and the parameter estimates from the data.**

**We agree that this distinction is crucial. We have revised the text throughout to be more specific, particularly for the covariance matrices used. We have also taken greater care to separate out covariance matrices of the real reference data set and those of the simulation (both population and sample covariances). Specifically, we now use** ${\hat{\boldsymbol{\Sigma}}}_{\boldsymbol{ref}}$ **to refer to the sample covariance matrix of the reference data (after the normalizing transform),** $\boldsymbol{\Sigma}_{\boldsymbol{sim}}$ **to refer to the population covariance matrix of the simulated data, and** ${\hat{\boldsymbol{\Sigma}}}_{\boldsymbol{sim}}$ **to refer to the sample covariance matrix of a simulated data set with the same number of samples as the reference data set. The plain** $\boldsymbol{\Sigma}$ **notation is now used just when referring to the parameter of the multivariate normal distribution or otherwise speaking in general terms without reference to any data set or simulation.**

3. **The authors seem to use the notation Z to represent different objects. Sometimes it is a p-dimensional random vector. Sometimes it is a p by n matrix.**

**We apologize for this inconsistency. We now use** $\boldsymbol{z}$ **to refer to the column vector and** $\boldsymbol{Z}$ **to refer to the matrix.**

4. **In the Methods Section, the authors did not give enough context before describing the methods. I suggest they move this part to the front of the article or include more context.**

**We have substantially revised the exposition of these methods in the article. Now, the Results section starts with a more conceptual overview and motivation, including new figures (Figure 1) with a diagram and demonstration of the difficulty of choosing the covariance matrix as better motivation. The detailed description of Gaussian copula simulations is moved to Methods, immediately before the description of our methods, giving them better mathematical context in addition to the conceptual context provided in Results.**

5. **When describing the PCA method, the authors state V = Z^T Z / (n-1) is the covariance matrix of Z. However, this is incorrect if Z is p by n.**

**Thank you for catching this error. We have corrected it and also switched to using the** ${\hat{\boldsymbol{\Sigma}}}_{\boldsymbol{ref}}$ **terminology instead of V for this matrix.**

6. **It is unclear what is the meaning of “reference dataset”**

**We use that term to refer to a real data set which we want to use as a reference while generating simulated data, with the goal that the simulated data should look like the reference data set. We have made this clearing in the manuscript by the following description to the start of Results:**

**We start with a reference dataset X given by an p x n data matrix of p features measured in each of n independent samples, which could be any real dataset of interest. Our goal is to generate a simulated dataset that looks, at least on average, the same as this reference dataset.**

2. **The authors described three methods and included all of them in their R package. However, the last method from another R package proposed by other people. It doesn’t seem appropriate to directly use the same method and repackage it to be one’s own.**

**We disagree with this assessment for several reasons. First, we appropriately cite the method that we build upon and refer to it by name throughout. Second, the corpcor package produces covariance matrix estimations, while our package produces simulated data. These are distinct use cases. Third, the corpcor package returns the estimated covariance matrix as a dense, full-rank matrix which means that simulating data from its output requires the use of a Cholesky or eigenvector decomposition of the covariance matrix. This is the computationally costly step that we seek to avoid. Instead, our method directly produces the decomposed form (diagonal plus low-rank) which makes these more costly procedures unnecessary and enables the efficient generation of random samples. This means that our method could not have been implemented by simply calling their method’s current implementation, even though mathematically they are the same covariance matrices. To our knowledge, the use of this decomposed form for efficient random sampling is entirely novel and in no way redundant with the existing corpcor package. We have substantially rewritten the paper to better emphasize this novel contribution and have added benchmarking to demonstrate the large differences our efficient sampling process creates in run-time and memory use.**

3. **For DESeq2 application, Figure 2 shows the result. However, the performances across independence, PCA, Wishart, and corpcor seem to be the same, with the authors’ proposed methods have more variance. Therefore, it is unclear why the authors’ methods are better.**

**We apologize for being unclear here. The goal of this section is not to improve performance of DESeq2 or reduce variance. We believe that the comparisons to real data (which have been expanded to now include, in supplemental, all the data sets used for the applications) demonstrate the superiority of our methods to simulations assuming independence. Therefore, we expect that our simulations are more realistic and better capture the true behavior of DESeq2 in real applications. When we observe higher variance of DESeq2 results in our methods than in the independent simulations, we believe that this better measures the variance that DESeq2 achieves in real applications. Our conclusion is then simply that benchmarking of DESeq2 must likewise use non-independent simulated data in order to capture the true behavior of DESeq2.**

4. **For CYCLOPS application, the authors did not give enough explanations on why corpcor is worse than PCA and Wishart.**

**Our hypothesis is that the performance of CYCLOPS in this dataset is driven by the number of eigengenes, which in turn is driven by the amount of correlation present in the simulation. To address this and other concerns, we have added Supplemental Figure 3, which compares the simulated datasets to the real reference dataset for this experiment. This demonstrates that corpcor had the lowest levels of correlation, Wishart method had somewhat higher, and PCA had the most (above even the real dataset). This tracks exactly with the observed number of eigengenes (higher correlation leading to fewer eigengenes used). We have added the following text for clarification in the CYCLOPS section:**

**Notably, the number of eigengenes and accuracy tracked both with each other and with the amount of gene-gene correlation (see Supplemental Figure 3 d) across the methods.**

5. **In general, the authors did not give guidance on how to choose among their three described methods.**

**We apologize for the omission of this. We have added the following text to Discussion:**

**We observe that our PCA method generally overshoots the level of correlation in the real data set. The corpcor method typically undershoots. The spiked Wishart method also undershoots but less so than the corpcor method and therefore we believe represents the best starting point for users. However, we emphasize that in any application, the users should compare their simulated data to the reference data set and this can be used to determine the best method for the specific application at hand, which could vary depending on data set.**

6. **There are many types of omics datasets. Do their methods apply to all of them?**

**We have now added a demonstration of our methods on another omics data type: a plasma metabolomics dataset from NMR, see Figure S1. We apply a normal marginal distribution to simulate these data sets (after a log transformation of abundance values). This demonstrates our methods on a very different example data set. Of course, there remain many other types of omics that have not been demonstrated, which is a limitation of our study, but we believe it is widely applicable.**

7. **The authors have only compared their methods to independence. It is not surprising the methods that consider correlations have better performance. The authors should also compare their method to not other methods that consider correlations.**

**Thank you for raising this important point. We now include comparisons to SPsimSeq in the figures where we compare fidelity to the reference real data sets (Figure 2, S2, and S3). This shows that SPsimSeq performs comparably to our own, particularly similarly to our PCA method. We also added benchmarking comparisons to other methods, see next response and Figure 1c-d.**

8. **The authors state that their methods can compute the correlations quickly. They should describe this advantage in more details and include some examples to illustrate.**

**We agree that this was a major oversight and have increased the emphasis on this advantage in our exposition. We have also added benchmarking (Figure 1c-d) showing run-time performance and memory usage of our methods, SPsimSeq, multivariate normal random values using the mvrnorm function, and a vine copula method which has been used in two relatively recent simulators. This benchmarking demonstrates a large advantage in run-time and memory usage for our methods (100x run-time and 20x memory). Memory use in particular is a major barrier for running alternatives on large data sets. The vine copula approach does not scale at all to the gene counts we use in these benchmarks, so we do not run it on the example applications. Similarly, the mvrnorm data is insufficiently realistic for actual application due to assuming a normal distribution**

**We also include more theoretical details about how the random sampling works and why it is faster, see Methods:**

**Generating data for** $\boldsymbol{\Sigma}_{\boldsymbol{sim}}$ **of this form is highly efficient. We use two basic facts about the multivariate distribution. First, if A is a matrix and** $\boldsymbol{u \sim N(0,}\boldsymbol{\Sigma}\boldsymbol{)}$ **then** $\boldsymbol{Au \sim N(0, A}\boldsymbol{\Sigma}\boldsymbol{A}^{\boldsymbol{T}}\boldsymbol{)}$**. Second, if** $\boldsymbol{u \sim N(0,}\boldsymbol{\Sigma}_{\boldsymbol{1}}\boldsymbol{)}$ **and** $\boldsymbol{v \sim N(0,}\boldsymbol{\Sigma}_{\boldsymbol{2}}\boldsymbol{)}$**, then** $\boldsymbol{u+v \sim N(0,}\boldsymbol{\Sigma}_{\boldsymbol{1}}\boldsymbol{+}\boldsymbol{\Sigma}_{\boldsymbol{2}}\boldsymbol{)}$**. Therefore, given matrices D, U, W and** $\boldsymbol{u \sim N(0,}\boldsymbol{I}_{\boldsymbol{k}}\boldsymbol{)}$ **and** $\boldsymbol{v \sim N(0,}\boldsymbol{I}_{\boldsymbol{p}}\boldsymbol{)}$ **(where** $\boldsymbol{I}_{\boldsymbol{k}}\boldsymbol{,}\boldsymbol{I}_{\boldsymbol{p}}$ **are the** $\boldsymbol{k\times k, p\times p}$ **identity matrices), then** $\boldsymbol{Dv+UW u \sim N}\left( \boldsymbol{0,}\boldsymbol{D}^{\boldsymbol{2}}\boldsymbol{+U}\boldsymbol{W}^{\boldsymbol{2}}\boldsymbol{D}^{\boldsymbol{T}} \right)\boldsymbol{=N(0,}\boldsymbol{\Sigma}\boldsymbol{)}$ **as desired.**

**In contrast, when given an arbitrary** $\boldsymbol{\Sigma}$ **to generate a random value in** $\boldsymbol{N}\left( \boldsymbol{0,}\boldsymbol{\Sigma} \right)$ **one must compute a matrix V such that** $\boldsymbol{V}\boldsymbol{V}^{\boldsymbol{T}}\boldsymbol{=}\boldsymbol{\Sigma}$**. Then, using** $\boldsymbol{v \sim N}\left( \boldsymbol{0,I} \right)$**, we have** $\boldsymbol{Vv \sim N}\left( \boldsymbol{0,}\boldsymbol{\Sigma} \right)$ **as desired. However, computing V is done using a Cholesky decomposition or eigenvector decomposition, both of which are computationally expensive when** $\boldsymbol{\Sigma}$ **is large.**
